# Emergency response for recently isolated Foot and Mouth Disease virus type A Africa in Egypt 2022

**DOI:** 10.1038/s41598-025-88906-4

**Published:** 2025-02-06

**Authors:** Mohamed Samy Abousenna, Heba A. Khafagy, Amal Abd El Moneim Mohamed, Sara E.A. El Sawy, Fady Abd El Mohsen Shasha, Darwish Mahmoud Darwish, Nermeen G. Shafik

**Affiliations:** Central Laboratory for Evaluation of Veterinary Biologics, Agricultural Research Center, P.O. Box 131, Cairo, 11381 Egypt

**Keywords:** Foot-and-Mouth Disease, FMDV vaccine, Vaccine matching, Challenge test, VNT, Disease outbreak, Inactivated vaccines, Inactivated vaccines, Virology

## Abstract

Foot and mouth disease (FMD) is a highly contagious viral infection affecting cloven-hoofed ruminants, leading to significant economic losses. In 2022, Egypt faced a severe outbreak of Foot and Mouth Disease (FMD) caused by the A/Africa/G-IV variant. This study assessed the efficacy of local and imported FMDV vaccines (A Iran-05 lineage) against this new variant using in vitro and in vivo methods. Sera from vaccinated calves showed inadequate cross-protection, with mean r1-values of 0.235 and 0.243 for local and imported vaccines, respectively. Challenge tests indicated low protection levels (20% and 40%) against A/Africa/G-IV compared with A/Iran/05. Current vaccines were deemed ineffective, prompting a formulation update incorporating the variant. The modified vaccine is now deployed in proactive vaccination efforts to address the evolving FMD outbreak.

## Introduction

Foot and Mouth Disease (FMD) is a highly contagious viral infection with significant economic consequences, particularly among cloven-hoofed ruminants. This disease affects various livestock species, including cattle, swine, sheep, and goats^1^, the causative agent of Foot and Mouth Disease (FMD) is an aphthovirus belonging to the Picornaviridae family. This virus is characterized by its nonenveloped, single-stranded structure and possesses a positive-sense RNA genome of approximately 8500 bases. Notably, the virus displays both antigenic and genotypic distinctions, enabling its classification into seven serotypes^2,3^.

Foot-and-mouth disease (FMD) presents a global challenge with seven distinct viral strains (A, O, C, SAT1, SAT2, SAT3, Asia1) requiring specific vaccines for each^4^. The insidious nature of the virus allows detection in milk and semen before clinical signs such as fever and blistering of the oral cavity, teats, and interdigital spaces manifest^5^. While often nonfatal in mature animals, FMD poses a significant threat to young animals, causing myocarditis and substantial production losses. Recovered animals can become intermittent carriers, potentially triggering future outbreaks^6^. Egypt has a history of FMD serotype A, with the first case identified in the 1960s. A major outbreak in 2006 revealed similarities between the Egyptian strain and those found in East Africa^7^. A different strain, A-Iran05-08, which was originally detected in Iran, was also documented in Egypt between 2010 and 2015^8^. Since 2012, the African type-G-IV variant has been found in Egypt, with additional detections reported in 2016, 2018, and 2020^9^. Vaccination plays a crucial role in disease control, especially in the context of FMD.

The vaccine should include multiple serotypes of FMDV to ensure protection against prevailing endemic field strains in Egypt^10^. Recently, isolated FMDV variants in Egypt have demonstrated significant genetic variation from locally used vaccine strains, especially in the major antigenic sites of the VP1 region. The circulating FMDV strains in Egypt in 2020 belong to serotype A, Africa topotype G-IV, and exhibit substantial genetic variation in the VP1 region from previously reported strains in Egypt, with a mean value of 93.7%. Notably, strain A/Africa/G-IV is not included in the currently deployed local and imported vaccines in Egypt, posing a potential risk for future FMD outbreaks and an increased likelihood of further genetic mutations^9^.

The current locally produced and imported vaccines urgently require reevaluation to mitigate the risk of recurring FMDV outbreaks^11^. Consequently, in the present study both in vitro and in vivo studies were conducted to assess the extent to which the current vaccines can protect calves against the recently isolated strain.

## Materials and methods

### Virus and cells

The FMDV A/Africa/G-IV variant in Egypt was isolated and detected at the Animal Health Research Institute (AHRI) through reverse transcription real-time polymerase chain reaction (RT-qPCR) and enzyme-linked immunosorbent assay (ELISA)^12^. Subsequently, AHRI officially provided the virus to the Central Laboratory for Evaluation of Veterinary Biologics (CLEVB), and was designated for the assessment of existing inactivated FMDV vaccines. This virus was adapted to the baby hamster kidney cell line (BHK-21 cells), attaining a titer of 10^4^ TCID_50_/mL, which was intended for use in the virus neutralization test (VNT) and as a virulent strain for the challenge test involving experimentally vaccinated calves.

The FMDV A/EGY/1/2012 (A Iran-05) strain was obtained from the Strain Bank Department at CLEVB, specifically for the purpose of evaluating the FMDV vaccine in accordance with WOAH^13^.

BHK-21 cells were provided by the FMD vaccine production Department at the Veterinary Serum and Vaccine Research Institute (Abbassia, Cairo, Egypt).

## Vaccine

CLEVB meticulously selected two FMD vaccine batches (*n* = 2) with a focus on their outstanding potency results. These batches were deliberately chosen to provide comprehensive representation, encompassing all prevalent Foot and Mouth Disease Virus (FMDV) serotypes circulating in Egypt. The meticulous selection process aimed to ensure a robust and representative evaluation of vaccine efficacy against the diverse FMDV strains present in the region.

Batch (1): Local commercial trivalent oil -inactivated FMDV vaccines were produced in Egypt and prepared from local isolate serotypes (O/EGY/4/2012, A/EGY/1/2012 and SAT2/EGY/2/2012).

Batch (2): The imported polyvalent oil -inactivated FMDV vaccine included O Manisa, O-3039, A Iran-05, A Saudi-95, Asia − 1 Shamir and SAT-2.

## Calves

Twenty-six calves of a native breed, aged between 6 and 8 months, were provided by the CLEVB, Agricultural Research Center (ARC). For experimental purposes, these animals were categorized into four groups and housed in distinct stables within the animal facility at CLEVB. All procedures were sanctioned by the Institutional Animal Care and Use Committee (IACUC) at the ARC.

These calves were previously screened for the presence of specific antibodies against FMDV A/Africa/G-IV using VNT (seronegative).

### Group (1)

Two calves were subjected to virus titration.

### Group (2)

Ten calves were vaccinated subcutaneously (S/C) with one field dose according to the manufacturer’s instructions for the previously evaluated local commercial FMDV vaccine (Batch 1).

### Group (3)

Ten calves were vaccinated with one field dose of S/C according to the manufacturer’s instructions for the previously evaluated imported FMDV vaccine (Batch 2).

### Group (4)

Four calves were included in the nonvaccinated group (control positive for the challenge test).

The design of the animal experiments in this study adhered to the guidelines outlined in the studies by Abousenna^10^and Nermeen^14^.

## Virus titration in calves’ tongue

The infectivity titration of the FMDV A/Africa/G-IV variant, intended for use in the challenge test, was performed on the tongues of calves in Group 1. The procedure was conducted according to the methodologies detailed by Dekker^15^and Abousenna^11^, which involve specific techniques for inoculating and assessing viral infectivity in the target tissue. This careful adherence to established protocols ensures the reliability and accuracy of the titration results. The virus titer was subsequently computed and expressed as the log_10_ Bovine Infective Dose (BID_50_/mL) according to Karber^16^.

## Virus neutralization test

The examination was conducted on BHK-21 cells utilizing the microtiter neutralization technique as described by Ferriera and WOAH [17. 18]. The VNT antibody titer was considered protective when it reached ≥ 1.65 log₁₀ TCID₅₀, providing a benchmark for vaccine efficacy.

### Challenge test

On the 28th day postvaccination, both groups of vaccinated calves (Groups 2 and 3) and the control group (Group 4) were relocated to the challenge room within the animal facility. Each vaccinated group was further subdivided into two subgroups, with five calves in each subgroup. One subgroup from each vaccinated group was challenged with the FMDV A/Africa/G-IV variant, whereas the other subgroups were challenged with the FMDV A/EGY/1/2012 (A Iran-05) strain. The challenge test procedures and interpretations were conducted according to WOAH [18. 19]. The protective level percentage was calculated at seven days post-challenge as the proportion of vaccinated calves that remained protected (i.e., showed no clinical signs) following the challenge. A protective level of ≥ 75% was considered indicative of effective vaccine performance.

The experimental animals subjected to the challenge test were provided with animal care and management, following the procedures outlined by Abousenna^11^.

## Determination of the serological relationship (r1- value)

On the 28th day following vaccination, blood sera were collected from the vaccinated animals (Groups 2 and 3) prior to the challenge. These sera were then subjected to testing against both the vaccine strain (FMDV A/EGY/1/2012 (A Iran-05)) and the field-isolated variant (FMDV A/Africa/G-IV) using the Virus Neutralization Test (VNT), The calculation and interpretation of the r1-value were performed in accordance with WOAH guidelines^18,20,21^.

## Results

Commercial FMDV vaccine batches (*n* = 2) were evaluated at CLEVB for potency against FMDV serotype A/EGY/1/2012 (A Iran-05) using VNT and a challenge test. Both vaccine batches (1 and 2) demonstrated satisfactory results, with neutralizing antibody titers of 2.04 and 2.46 log_10_, respectively, as detailed in Table 1.

The infectivity titration of FMDV A/Africa/G-IV variant in calves’ tongues (group 1) yielded a titer of 10^5^ BID_50_/mL. In both the vaccinated groups (2 and 3) with inactivated FMDV vaccine batches (1 and 2), and the non-vaccinated control calves group (4), which were bled before challenge for antibody titer screening at the 28th day post-vaccination using VNT, neutralizing antibody titers of 0.48, 0.6, and 0.15 log_10_ were observed, respectively, as indicated in Table 1.

In the challenge test, the protection levels were 20%, 40%, and 0% against FMDV A/Africa/G-IV variant for the vaccinated groups (2 and 3) and the control group (4), respectively. Moreover, the protection levels were 80%, 100%, and 0% against FMDV serotype A/EGY/1/2012 (A Iran-05) for the vaccinated groups (2 and 3) and the control group (4), as indicated in Table 1.

At seven days post-challenge, protection and positivity (FMDV characteristic lesions) were assessed. In group 2, vaccinated with the local vaccine (Batch 1), one out of five animals (20%) was protected against FMDV A/Africa/G-IV, showing no lesions, while four out of five animals were positive for FMDV A/Africa/G-IV, exhibiting lesions. For FMDV A/EGY/1/2012, four out of five animals (80%) were protected, showing no lesions, while one calf was positive, exhibiting lesions.

In group 3, vaccinated with the imported vaccine (Batch 2), two out of five animals (40%) were protected against FMDV A/Africa/G-IV, showing no lesions, while three out of five were positive, exhibiting lesions. All five animals (100%) were protected against FMDV A/EGY/1/2012, showing no lesions.

In the unvaccinated control group (group 4), none of the animals were protected against either virus strain, and all developed lesions. The lesions ranged from mild to moderate on the tongue and severe on all four feet.

The serological relationship (r1-value) was determined using a virus neutralization test (VNT) to evaluate cross protection of the sera from vaccinated calves in groups 2 and 3 against the isolated FMDV A/Africa/G-IV variant. As shown in Table 2, the r1-values were 0.235 and 0.243 for groups 2 and 3, respectively, indicated that the tested vaccine did not provide adequate protection against the field isolate.

**Table 1:** Evaluation of the humoral immune response and protection level of vaccinated calves receiving local and imported FMDV vaccines.

Group of Calves	Vaccine Batch	FMDV Strain (Complete Nomenclature)	VNT Antibody Titer (Log_10_ TCID_50_)	Mean VNT Titer (Log_10_ TCID_50_)	Protective Level (Percentage %)
Group 2	Batch (1) Local Vaccine	A/Africa/G-IV	Calf 1: 0.6	Mean: 0.48	20%
			Calf 2: 0.3		
			Calf 3: 0.6		
			Calf 4: 0.3		
			Calf 5: 0.6		
		A/EGY/1/2012	Calf 6: 1.8	Mean: 2.04	80%
			Calf 7: 2.1		
			Calf 8: 1.8		
			Calf 9: 2.4		
			Calf 10: 2.1		
Group 3	Batch (2) Imported Vaccine	A/Africa/G-IV	Calf 1: 0.6	Mean: 0.6	40%
			Calf 2: 0.3		
			Calf 3: 0.3		
			Calf 4: 0.6		
			Calf 5: 0.6		
		A/EGY/1/2012	Calf 6: 2.1	Mean: 2.46	100%
			Calf 7: 2.4		
			Calf 8: 2.7		
			Calf 9: 2.7		
			Calf 10: 2.4		
Group 4	Unvaccinated (Control)	A/Africa/G-IV	Calf 1: 0.3	Mean: 0.15	0%
			Calf 2: 0.0		
		A/EGY/1/2012	Calf 3: 0.3	Mean: 0.3	0%
			Calf 4: 0.3		

* Cut-off titre for evaluating immunological protection afforded by vaccination ≥ 1.65 log_10_ TCID_50_.

** Protection level (%) of challenge test ≥ 75%.

**Table 2:** r1-value of FMDV A Africa and A Iran-05 using VNT.

Type of vaccine	Local Vaccine ( batch 1)	**I**mported Vaccine (batch 2)
**r1 value**	0.235	0.243

R < 0.3 indicated highly significant antigenic variation from the vaccine strains and another vaccine strain should be chosen.

R > 0.3 demonstrated that the vaccine and field strains are sufficiently similar and the vaccine would provide good protection.

## Discussion

During the 2022 FMDV outbreak, Egyptian authorities swiftly implemented emergency measures and stringent control precautions to address the situation. Prioritizing rapid isolation and identification of FMDV, a prompt response was initiated for efficient containment of the outbreak. In this study, we investigated the effectiveness of the existing local and imported commercial vaccines against the recently isolated FMDV A/Africa/G-IV variant through the Virus Neutralization Test (VNT) and the challenge test^11,19,22^. The effective management of FMDV disease is contingent upon the availability of vaccines, chosen based on genome alignment, epidemiological data, and the serological cross-reactivity of bovine postvaccinal serum with prevalent viruses. Additionally, ensuring the adequacy of vaccine doses and maintaining good quality and potency are equally crucial^23^. In Egypt, polyvalent inactivated vaccines are currently utilized for the prevention of FMDV infection. However, the observed genomic variability in recent circulating field isolates raises concerns regarding their alignment with the vaccine strain^9^.

In the presented study. Calves from groups 2 and 3 were subcutaneously inoculated with FMDV vaccines. In calves immunized with the local commercial vaccine, the humoral immune response against the FMDV isolate field variant A/Africa/G-IV and FMDV A/EGY/1/2012 (A Iran-05) strain was determined by VNT, revealing titers of 0.48 and 2.04 log_10_, respectively. In contrast, the humoral immune response of the imported vaccine against the FMDV isolate field variant A/Africa/G-IV and FMDV A/EGY/1/2012 (A Iran-05) strain, as assessed by VNT, showed titers of 0.6 and 2.4 log_10_, respectively, meeting the minimum protective virus-neutralizing antibody titer of ≥ 1.65 log_10_^18^.

The serological relationship, represented by the r1-value, of the recently isolated FMDV A/Africa/G-IV variant was examined and computed. According to WOAH standards, during neutralization, r1-values exceeding 0.3 signify close antigenic correspondence between the vaccine strain and the field isolate, suggesting the potential for the vaccine strain to provide cross-protection against the field strain. Conversely, r1-values less than 0.3 indicate a lack of cross-protection. The calculated r1-values were 0.235 and 0.243 for the local and imported vaccines, respectively. This result aligns with the findings of Shahein^12^, who compared the isolated strains with the prevailing vaccine strains, revealing genetic identities of 84.55% ± 0.82% and 82.99% ± 0.8% with the A Iran-5 Egypt-2012 and Iranian-2005 strains, respectively. Notably, there was a 9.3% divergence from the formerly isolated local vaccine strain in 2020. Considering the genetic identity and similarity of the strains, other investigations have proposed a system for distinguishing FMDV strains using the virus neutralization reaction, emphasizing the importance of considering both the statistical and biological significance of the observed r1- values^24^.

The results concerning the protective efficacy of vaccine batches (1 and 2) against FMDV A/Africa/G-IV variant indicated protection levels of 20% and 40%, respectively. In contrast, the challenge test results for FMDV A/EGY/1/2012 (A Iran-05) revealed higher protection rates of 80% and 100%. According to the international guidelines provided by the WOAH^18^, a minimum effectiveness of 75% is recommended for routine vaccines. A similar study exploring the cross-protective potential of an A/G-VII emergency vaccine revealed that, despite its formulation at 43 (95% CI 8–230) PD50/dose as determined during homologous challenge, it did not provide adequate protection against viruses from the A/IRN/05 lineage. Consequently, while the A/G-VII vaccine strain cannot substitute for the A/IRN/05 vaccine strain, it may be considered an additional strain for incorporation in vaccines and antigen banks^25^.

## Conclusion

The current inactivated FMDV vaccines, whether locally produced or imported and specifically designed for the A Iran-05 lineage, have proven ineffective against the prevailing field isolate A/Africa/G-IV. In response to this challenge, the recommendation was made to enhance the existing vaccines by integrating the isolated variant alongside the current formulation. The vaccine manufacturer promptly responded by adjusting the formulation to include the isolated variant. The modified vaccine is now actively utilized in vaccination campaigns, serving as a proactive measure to prevent and manage the outbreak more effectively.

## Data Availability

“All data generated or analyzed during this study are included in this published article”.
